# Oxidative Stress Is Predominant in Female but Not in Male Patients with Autoimmune Thrombocytopenia

**DOI:** 10.1155/2014/720347

**Published:** 2014-01-06

**Authors:** Julian Kamhieh-Milz, Abdulgabar Salama

**Affiliations:** Institute for Transfusion Medicine, Charité University Medicine, Augustenburger Platz 1, 13353 Berlin, Germany

## Abstract

As the involvement of oxidative stress (OS) in autoimmune thrombocytopenia (AITP) has been reported, a fast and rapid test for the reliable measurement of OS and antioxidant capacities (AOCs) might be a useful tool in extending current diagnostic possibilities. The free oxygen radical test (FORT) and free oxygen radical defence (FORD) assay (Callegari, Italy) are easy to perform and reliable, with results available within 15 minutes. Thirty-seven AITP patients and 37 matched healthy individuals were included in this study. All participants responded to a standard questionnaire provided by these assays. Female patients with AITP were observed to demonstrate significantly higher OS in comparison to female controls (*P* = 0.0027) and male AITP patients (*P* = 0.0018). The AOCs were not reduced in patients with AITP (*P* = 0.7648). Correlation of OS with platelet count identified a weak positive correlation (*P* = 0.0327, Spearman *R* = 0.4672). The questionnaire revealed that ITP patients in comparison to healthy controls are more stressed, feel exhausted and fatigued, and eat a healthier diet. In conclusion, OS is predominant in female but not in male patients with AITP suggesting gender-specific differences in the pathomechanisms of AITP. Identification of patients with high levels of OS might be beneficial in the management of AITP.

## 1. Introduction

Primary autoimmune thrombocytopenia (AITP) is an acquired autoimmune disorder defined by isolated thrombocytopenia and exclusion of other causes [1, 2]. The triggering event for AITP remains unknown [3]. The dominant clinical symptoms are petechia and bleeding, which generally correlate with the severity of thrombocytopenia [4]. A normal platelet count in a healthy individual is between 150 and 450 × 10^9^ platelets/L. Most AITP patients are asymptomatic in the presence of platelet counts above 50 × 10^9^ platelets/L [5]. The clinical manifestation of AITP is extremely variable [4] and, to date, there are no AITPspecific diagnostic markers or disease-specific therapeutic treatments available [6]. AITP embodies a prototype of a B-cell mediated autoimmune lymphoproliferative disease. Antiplatelet autoantibodies are detectable in 50–60% of patients and are thus of diagnostic relevance [7]. Autoreactive T-cells also play a significant role in the cross talk between antigen presenting cells and autoantibody producing B-cells [8].

Oxidative stress (OS) is termed as an imbalance between the systemic manifestation of reactive oxygen and nitrogen species (ROS and RNS, resp.) and an individual's ability to detoxify these highly reactive species to prevent and/or repair damaged components. All types of biological molecules such as DNA, proteins, and lipids can be targeted by ROS and RNS. In target proteins, the nitration and other OS-related posttranslational modifications of amino acids take place. These include the alteration of the physical and chemical structure of target proteins causing oxidation of side-chain groups, protein scission, cross-linking, unfolding, and the formation of new reactive groups and thus followed by loss of protein function, resulting in cytotoxic by-products and/or protein aggregates, and may trigger the generation of cryptic and/or neoepitopes [9–12]. The later has great implication on the role of OS and autoimmunity leading to B- and T-cell dysregulation and the generation of autoantibodies [13–16]. This highlights the pleiotropic impact of OS on autoimmunity. Few studies have addressed the involvement of OS in AITP. The very first report of oxidative dysregulation in neutrophils of AITP patients was demonstrated in 1984 by Ohno et al. [17]. In the 1990s, ascorbic acid, a well-known antioxidant, was used for the treatment of AITP, with 7 of 11 (63.6%) patients positively responding [18]. However, several groups failed to reproduce these results [19–21]. Two previous studies on paediatric AITP patients investigated antioxidant parameters including serum MDA, total antioxidant capacity (AOC), and total oxidant status, which were found to be significantly elevated in acute and chronic AITP [22]. Gene expression profiles of peripheral blood mononucleated cells (PBMCs) revealed OS-related pathways to be most significant in chronic AITP [23]. In adult AITP patients, elevated levels of plasma and erythrocyte malonyldialdehyde (MDA), reduced erythrocyte glutathione, and ascorbic acid have also been reported [24]. Recently, Jin and coworkers reported on serum nitrogen monoxide (NO), glutathione (GSH), glutathione disulphide (GSSG), MDA, total antioxidant status, total oxidant status, superoxide dismutase, and catalase and glutathione peroxidase levels in adult ITP patients, with strong correlations observed between these parameters and platelets counts [25]. We were the first to investigate the intracellular generation of ROS in platelets in order to characterize platelet AOCs and identified a reduced AOC of platelets from AITP patients compared to healthy donors, suggesting that platelets are under the attack of a systemic OS [26]. The aim of this study was to investigate systemic OS and AOCs of AITP patients. As free oxygen radicals are rapidly metabolized *in vivo*, their evaluation represents an extremely difficult task. The free oxygen radicals test (FORT) and free oxygen radicals defence (FORD) assays (Callegari, Parma, Italy) overcome these issues and allow for a fast and rapid determination of both the total blood peroxide concentration and total AOC in a highly stable, accurate, and reproducible manner.

## 2. Materials and Methods

### 2.1. Subjects

Thirty-seven patients with AITP (*n* = 34 primary, *n* = 3 secondary) and 37 healthy blood donors (controls) matched in respect to age, sex, and body mass index (BMI) were included in this study. AITP was diagnosed in accordance with the guidelines of the American Society of Hematology [27]. Demographic data and clinical parameters are presented in Table 1. Approval from the institutional ethics review board (EA2_130_09) and informed consent from all participants in this study were obtained.

### 2.2. FORT (Free Oxygen Radicals Test): Oxidative Stress/Free Radicals Test

The FORT colorimetric assay (Callegari, Parma, Italy) is based on the ability of transition metals, such as iron, to catalyze the breakdown of hydroperoxides (ROOH) into derivative radicals, according to the Fenton reaction. When 20 *μ*L of capillary blood is dissolved in an acidic buffer provided by the manufacturer (R2), the hydroperoxides react with the transition metal ions liberated from the proteins in the acidic medium and are converted to alkoxy (RO^•^) and peroxy (ROO^•^) radicals (reactions A and B). The radical species produced by the reaction interact with an additive (phenylenediamine derivative, 2CrNH_2_) that forms a colored solution (reaction C). The RBCs are then spun down (~960 ×g, 60 s) and the cuvette is placed into the spectrophotometer (FORMplus, Callegari). Following six min at 37°C (standardized temperature for accurate and reproducible measurements), the color is estimated at 505 nm (linear kinetic-based reaction). The intensity of the color correlates directly with the quantity of radical compounds and with the hydroperoxide concentration accordingly to the Lambert-Beer law:

(1) (A)  R-OOH+Fe2+⟶RO•+OH−+Fe3+(B)  R-OOH+Fe3+⟶ROO•+H++Fe2+(C)  RO•+ROO•+2CrNH2⟶ROO−+RO−+[2CrNH2+•]

Results are expressed as FORT U (units), whereby 1 FORT U is equivalent to 0.26 mg/L H_2_O_2_. Oxidative stress classifications are as follows: no oxidative stress < 230 FORT U, intermediate 230–310 FORT U, oxidative stress 310–400 FORT U, and strong oxidative stress > 400 (upper detection limit is 600 FORT U).

### 2.3. FORD (Free Oxygen Radicals Defence): Antioxidant Capacity/Defence Test

The FORD test (Callegari, Parma, Italy) is based on the decrease in absorbance that is proportional to the total AOCs of blood serum in accordance to the Lambert-Beer law. In the presence of an acidic buffer with a pH of 5.2 (S2) and a suitable oxidant (FeCl^3+^) (S3), a stable and colored radical solution is formed by the chromogen that contains 4-amino-N, N-diethylaniline sulfate. Fifty *μ*L of whole blood was obtained from the fingertip and transferred into an Eppendorf tube containing S1 solution. Our own observations revealed identical values when blood was stored in S1 solution for up to 30 min. Within the first measurement of the S2 solution containing 50 *μ*L of the S3 solution, the maximum color intensity develops (first reaction). The whole blood in the S1 solution is then spun down (1 min, ~960 ×g) and 100 *μ*L of the supernatant (serum) is transferred into the cuvette. Antioxidant compounds in the sample reduce the radical cation of the chromogen, quenching the color and producing a decoloration of the solution, which is proportional to their concentration. The decrease in absorbance is then measured photometrically at 505 nm at 37°C (second reaction). The absorbance values obtained for the samples are compared with a standard curve obtained using Trolox (6-hydroxy-2,5,7,8-tetramethylchroman-2-carboxylic acid), a permeable cell derivative of vitamin E commonly used as an antioxidant. So,

(2) first reaction:  Chromogen(no  color)+Fe2++H+⟶Chromogen•+(purple)second reaction:  Chromogen•+(purple)+AOH⟶Chromogen(no  color)+AO

The linearity range is from 0.25 to 3.0 mmol/L Trolox. The assay is usually completed within six minutes. Classification of the AOCs is as follows: good AOCs > 1.53 mmol/L Trolox and normal is between 1.07 and 1.53 mmol/L Trolox whereas values below 1.07 are considered as reduced AOCs.

A step-by-step schematic overview of the FORT and FORD assays is provided as supplementary material (Supplement 1 available online at http://dx.doi.org/10.1155/2014/720347). All reagents were provided as components of the test kits.

## 3. Statistical Analysis

Analyses were performed using GraphPad Prism version 5.0 for Windows (Graph Pad Software, San Diego, California, USA). Differences between controls and patients were examined using the Student's *t*-test or the nonparametric Mann-Whitney *U* test. The Spearman correlation coefficients for nonparametric variables were used to investigate correlations with FORT and FORD results in respect to platelet count, age, and BMI.

## 4. Results

### 4.1. Investigation of Oxidative Stress and Antioxidant Capacities from AITP Patients and Healthy Controls

Thirty-seven thrombocytopenic patients and 37 healthy controls were investigated via the FORT and FORD assays. Controls were matched in regard to sex, age, and BMI with no statistically significant differences between patients and controls observed (*P* > 0.05) (Table 1).

**Figure 1:**
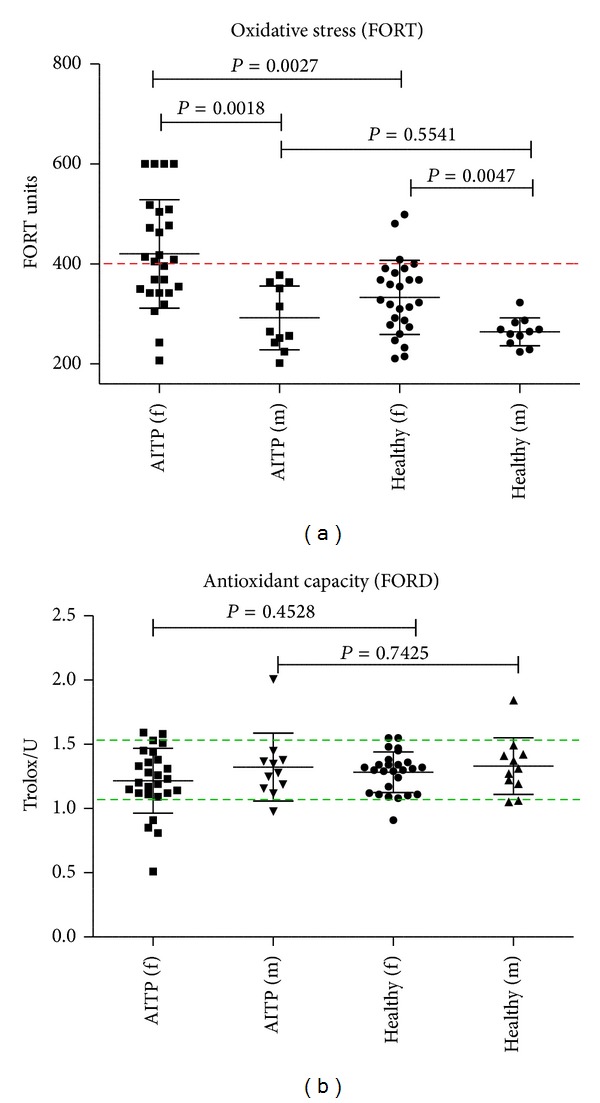
Comparison of oxidative stress and antioxidant capacities between AITP patients and healthy controls (FORT and FORD). Healthy females have higher OS than healthy males (*P* = 0.0047) requiring gender-specific comparisons. Women with AITP demonstrate a significantly higher degree of hydrogen peroxide in peripheral blood compared to healthy females (*P* = 0.0027) and male patients with AITP (*P* = 0.0018). No significant differences were observed in respect to AOCs. The broken line on the left indicates the threshold of strong OS and the area between the broken lines on the right indicates normal AOCs. Abbreviations: f = female, m = male, and AITP = autoimmune thrombocytopenia.

In accordance with our hypothesis, we found that AITP patients significantly demonstrate higher levels of OS in comparison to controls (*P* = 0.0047). No differences were observed with respect to the AOCs (*P* = 0.7648). Further analysis of the data revealed sex-specific differences in OS, with female controls notably demonstrating significantly higher concentrations of total peroxides in peripheral blood compared to male controls (*P* = 0.0047). The AOCs did not differ in the healthy control group in respect to gender (*P* = 0.7648). Comparison of patients with controls was repeated in a gender-specific manner (Figure 1). Although healthy female controls demonstrate elevated levels of OS, a significantly higher degree of OS was found for female patients with AITP (*P* = 0.0027). No significant difference between male AITP patients and male controls was found (*P* = 0.5541). Female patients with AITP demonstrated significantly higher OS than male patients with AITP (*P* = 0.0018).

### 4.2. Classification of Oxidative Stress

**Figure 2:**
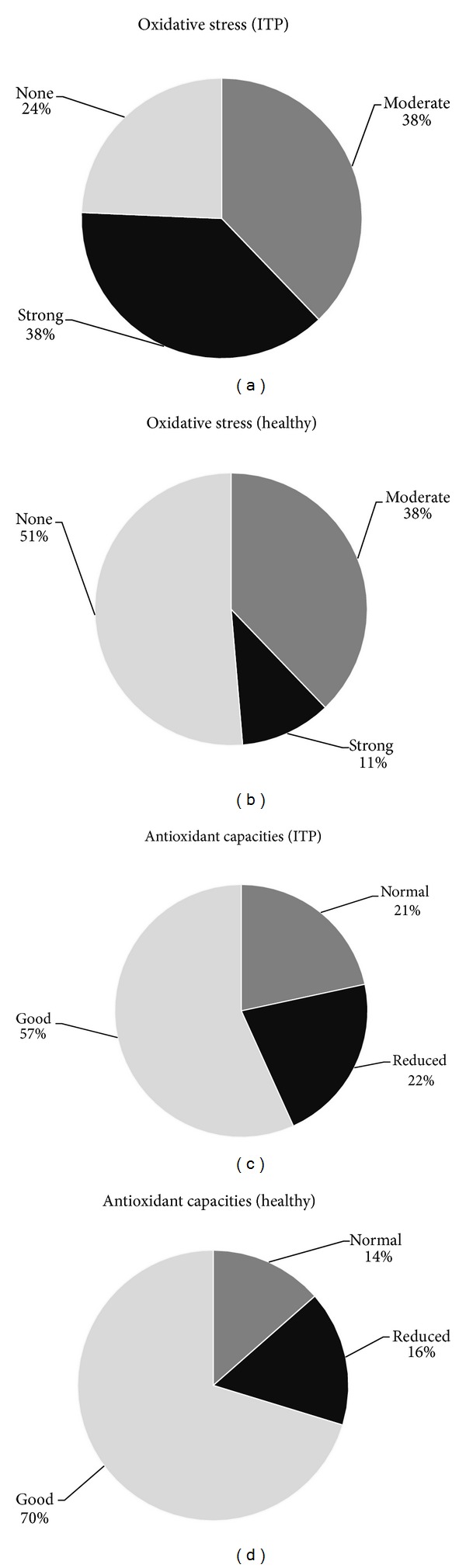
Distribution of oxidative stress levels and antioxidative capacities in AITP patients and healthy controls. Only 24% of AITP patients investigated were not observed to have oxidative stress (OS). More than one-third (38%) of the patients demonstrated hydrogen peroxide levels that are classified as strong OS. In the control group, only 11% of the controls have strong OS, whereas 51% were not observed to have OS. Although no significant difference between both groups was observed, healthy controls demonstrate generally better antioxidant capacities. In summary, oxidative damage is more predominant in patients with AITP (AITP = autoimmune thrombocytopenia).

Oxidative stress and AOCs were classified in accordance with the manufacturer's guidelines. As can be observed in Figure 2, 76% of patients demonstrated either moderate (230–310 FORT U) or strong OS (FORT U > 400). In contrast, only 49% of the healthy control group exhibited a moderate or strong increase. Interestingly, strong levels of OS were found in 38% of AITP patients in comparison to only 11% in the healthy control group. Although no statistically significant difference was discerned in respect to the AOCs, fewer AITP patients (57%) than controls (70%) demonstrated good AOCs (>1.53 Trolox/U). Twenty-two percent of AITP patients exhibited a reduced AOC (<1.07 Trolox/U) compared to 16% of the healthy controls.

There are several combinations of OS and AOCs. A strong OS does not simultaneously mean oxidative damage to biomolecules. Even a strong OS can occur in individuals with good AOCs, thus oxidative damage is less likely. Only a strong OS with simultaneously reduced AOC is considered as pathological. This combination was observed in five AITP patients (13.5%) and only one healthy control (2.7%) (Table 2).

### 4.3. Findings from the Oxidative Stress Questionnaire

All participants of this study filled a standard questionnaire developed from Micromedical which was provided with the FORT and FORD test kits. The questionnaire comprised of four main sections concerning A “general well-being and stress,” B “diet and lifestyle,” C “health and disease,” and D “drug intake.” The full list of questions and the frequency of each response are provided as supplementary material (Supplement 2).

**Figure 3:**
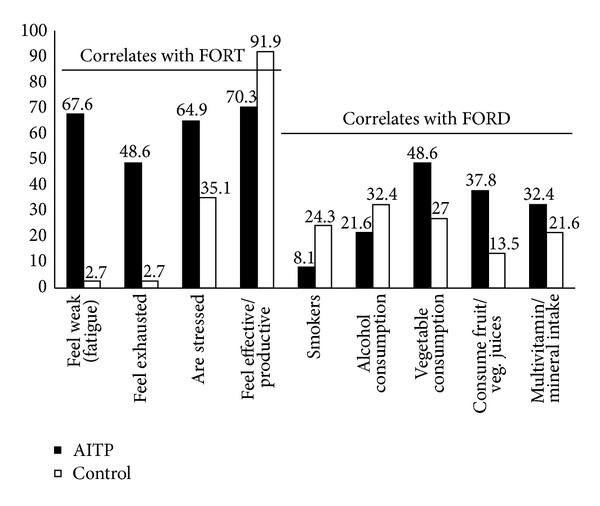
Bar blot analysis of the differences between AITP patients and controls revealed by the oxidative stress questionnaire. Results from the oxidative stress (OS) questionnaire revealed that approximately two-thirds of AITP patients suffer from fatigue. Fewer patients smoke cigarettes or drink alcohol, which is usually associated with an increase in OS. Patients were observed to have a more healthy diet in respect to the consumption of natural flavonoids and vitamins compared to controls (AITP = autoimmune thrombocytopenia).

The strongest differences between AITP patients and healthy controls are presented in Figure 3. Almost 70% of patients (*n* = 25) but only one from the control group felt fatigued, which is the most common symptom of AITP. Additionally, patients with fatigue demonstrate significantly higher OS than patients without fatigue (*P* = 0.0485). Almost 50% of AITP patients indicated that they feel exhausted (*n* = 18) in comparison to only one in the control group. Only 70.3% of the patients feel fully productive in comparison to 91.9% of the healthy controls. Altogether, almost double the number of AITP patients (*n* = 24, 64.9%) feel stressed in comparison to healthy controls (*n* = 13, 35.1%). This observation points towards strong differences between AITP patients in respect to OS and correlates well with the results revealed by the FORT assay. In general, AITP patients consume more fruits, vegetables, salads, fruit and vegetable juices, and minerals and/or multivitamins, smoke less, and consume less alcohol compared to controls.

### 4.4. Correlation of Systemic Oxidative Stress and Antioxidant Capacities with Platelet Count

**Figure 4:**
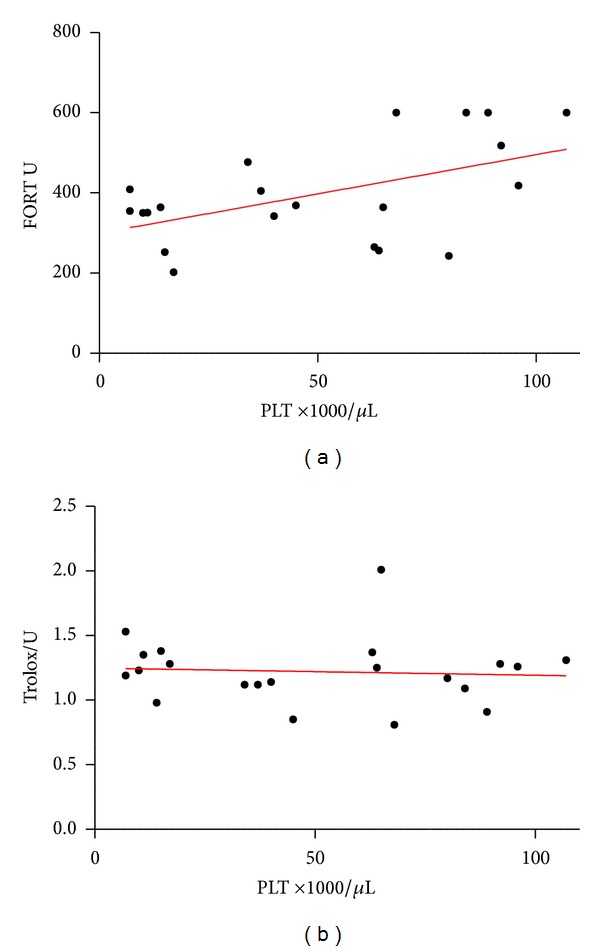
Correlation of platelet count with oxidative stress and antioxidant capacities. As platelet count is associated with disease severity, the correlation of oxidative stress (OS) and antioxidant capacities (AOCs) were investigated. A weak positive correlation was observed in respect to OS (*P* = 0.0327, Spearman *R* = 0.4672) whereas no correlation was observed with AOCs (*P* = 0.5307, Spearman *R* = −0.1449) (PLT = platelets).

Correlation of OS with platelet count identified a significant positive correlation (*P* = 0.0327, Spearman *R* = 0.4672), whereas no correlation was found between platelet count and AOCs (*P* = 0.5307, Spearman *R* = −0.1449; see Figure 4). However, a correlation with AOCs was observed in respect to BMI (*P* = 0.0048, Spearman *R* = 0.5904). Correlative analysis between OS and the corresponding AOCs was also performed and both parameters correlated well (*R* = −0.2892, *P* = 0.0124).

## 5. Discussion

In this study, systemic OS and systemic AOCs from 37 AITP patients and 37 age-, sex-, and BMI-matched healthy controls were investigated via the FORT and FORD assays. A direct comparison of AITP patients with healthy controls revealed AITP patients to have significantly higher OS levels and moderately but not statistically significantly decreased AOCs (Figure 1). This is the first report demonstrating that women with AITP have significant higher OS levels compared to healthy female controls and male patients with AITP (Figure 2), suggesting gender-specific differences in the pathophysiology of AITP.

There are several reports describing females to be predominantly afflicted with AITP [28–30]. This could be mediated by higher OS in females compared to male patients (*P* < 0.0018). Oxidative stress in women is also elevated in other pathological conditions such as coronary artery disease (CAD), neurodegenerative disease, atherosclerosis, and several types of cancer [31–34] and is associated with a higher risk of Parkinson's and Alzheimer's diseases [32, 33]. Gender is associated with differences in clinical presentation, onset, progression, and outcome of certain autoimmune diseases [35]. The role of gender and organ-specific autoimmunity is reviewed elsewhere [36]. In brief, females have stronger cellular and humoral immune reactions compared with males [37] and sex hormones are probably partly responsible for the higher occurrence of autoimmune disorders in females [38]. Sex hormones (e.g., estrogen) have been found to have immune modulating properties and influence the innate and adaptive immune cells [39, 40], antigen presentation [39, 40], cytokine secretion [35, 39, 41], and generation of autoantibodies [35] and is capable of stimulating autoreactive B-cells, promoting the escape of autoreactive cells from the mechanisms of immune tolerance [35]. Together with these findings, sex-specific differences in the pathophysiology of AITP might play a pivotal role and should be taken into consideration in future research.

The majority of patients investigated in this study (86.5%) do not suffer from a reduced systemic AOC (Figures 1 and 2). This suggests that a targeted reduction/inhibition of ROS generation rather than an antioxidant therapy is appropriate in treating AITP and might explain the failure of some studies which attempted to treat ITP with antioxidants [19–21]. Reducing free radical formation should thus be more efficient than trying to neutralize free radicals following their production. This can be realised by induction of hermetic pathways by caloric restriction, exercise which increases muscle levels of superoxide dismutase, glutathione peroxidase, and reduced glutathione, thermal stress which induces a heat-shock response, and increased expression of chaperones important to maintain the correct protein conformation and others. Collectively, this could be summarised as a lifestyle change and has been reviewed elsewhere [42].

In order to obtain additional information, all participants responded to a questionnaire in order to assess exposure to potential factors influencing OS and AOCs. The questionnaire comprised of more than 30 questions (see Supplement 2) with the strongest differences in the responses observed between AITP patients and controls, which is summarised in Figure 3. One of the most evident symptoms of AITP is fatigue. Almost 70% of patients (*n* = 25) suffered from fatigue, with only one control indicating such. One study investigating fatigue in adult primary AITP highlighted the clinical importance of this symptom [43]. In accordance with the findings of this study, chronic fatigue syndrome (CFS) occurs four times more frequently in women than in men, with CFS patients demonstrating signs of OS [44]. We can confirm that these findings as patients with fatigue demonstrated significantly higher OS than patients without fatigue (*P* = 0.0485). We observed that AITP patients eat more consciously (fruit, vegetables), drink relatively less alcohol, and smoke less in comparison to controls. The patients in this study might thereby compensate their AOC, thus resulting in no statistically significant differences between AITP and controls although a slight tendency of a decreased AOC was observed (Figure 2).

In order to determine whether OS is related to the disease activity, a potential correlation between OS and platelet counts was investigated. We observed a weak positive correlation of platelet counts and OS (*R* = 0.4687, *P* = 0.0321; see Figure 4). It is well-known that OS upregulates cortisol by the adrenal gland which increases platelet production as a stress response. This mechanism might explain the weak positive correlation of OS and platelet counts observed in this study. However, these findings are contradictory to the results of Jin and colleagues [25], who demonstrated reduced AOCs in adult patients with AITP, a strong negative correlation of platelet counts with total OS and a strong positive correlation of platelets counts with the total antioxidant status. This could be explained by the application of different assays. The FORT and FORD assays, which immediately measure OS and AOC, are clearly superior to ELISA in which serum samples are processed and frozen at −20 or −80°C prior to analysis. Furthermore, OS might rather be manifested in chronic AITP regardless of platelet count, which supports the findings of Zhang and colleagues who demonstrated OS-related pathways to be most significant in chronic (paediatric) AITP [23].

It remains unclear whether OS triggers autoimmunity or is the result of autoimmune dysregulation, which might explain the relatively slow progress in this area of research. For paediatric AITP, viral infections are known to be the triggering event for thrombocytopenia, whereas due to long incubation times, it is not possible to provide evidence that infections are also the cause of thrombocytopenia in adults with AITP. Nevertheless, asymptomatic infections are also assumed as the triggering event of adult AITP. As the generation of free radicals belongs to the host defence mechanisms against invading pathogens, it is highly likely that OS is present prior to autoimmune dysregulation. This supports the hypothesis that OS triggers autoimmunity. As ROS and RNS also act as a second messenger and are capable of activating transcription factors such as NF-*κ*B and AP-1, thereby upregulating cytokines, growth factors, and extracellular matrix proteins due to OS [45–47]. However, cytokines themselves can induce and enhance OS in a loop-like manner [48]. BAFF (B-cell activating factor) is a ligand of the Tumor Necrosis Family and is capable of preventing apoptosis of autoreactive B- and T-cells, and its overexpression results in the generation of autoantibodies [49, 50]. Interestingly, it was demonstrated that BAFF expression is increased under OS and can be downregulated by antioxidants [51, 52]. We were the first to report on increased BAFF serum levels in AITP [53]. This might underscore the importance of a systemic OS in human disease in general and, in particular, the pathophysiology of AITP. The impact of OS and autoimmunity is an avenue for further exploration. Reducing or preventing OS is crucial in order to avoid OS-mediated autoimmunity.

A systemic OS might also affect platelets in a direct manner. The observation of increased systemic OS in most of the AITP patients in combination with our previous report on decreased platelet AOCs, however, might point towards a direct destruction of platelets as a result of oxidative damage due to the induction of apoptosis [54]. In contrast, nitric oxide (NO) has the potential of inhibiting apoptosis in human platelets [55]. But reduced NO bioavailability in AITP patients has already been reported [56]. It is well-known that a shortened platelet life results in thrombocytopenia [57, 58]. In this respect, independently of autoimmune dysregulation and autoantibodies, increased free radical generation may also result in thrombocytopenia.

In conclusion, within this study, we identified that OS is predominant in female patients suggesting sex differences in the pathomechanisms of AITP. The AOCs were not observed to be decreased in AITP, potentially suggesting a prevention of ROS generation rather than antioxidant supplementation. However, reducing and/or preventing OS is crucial to avoid OS-mediated autoimmunity. To identify patients with OS, the FORT and FORD assays are easy to perform and reliable, with results available within 15 minutes, allowing for an immediate interpretation of results and prospective measures towards recommendations in preventing oxidative damage.

## Supplementary Material

Supplement 1 describes a step-by-step schematic overview of the FORT and FORD assays.
Supplement 2 entails a questionnaire developed from Micromedical completed by participants which was provided with the FORT and FORD test kits, and the frequency of each response. The questionnaire comprised of four main sections concerning A “general well-being and stress,” B “diet and lifestyle,” C “health and disease,” and D “drug intake”.

## Figures and Tables

**Table 1:** Demographic data and study design.

Demographic/clinical data	AITP	Controls	*P* value
Total	37	37	—
Male/female	11/26	11/26	—
Age	42.1 ± 17.0	41.0 ± 15.1	0.8294
Weight (kg)	70.0 ± 15.1	74.3 ± 15.7	0.2470
Height (cm)	169.6 ± 7.1	170.5 ± 9.4	0.6440
BMI	24.3 ± 4.8	25.4 ± 4.5	0.3315
Disease state: active/stable partial remission/complete remission	25/6/6	—	—
Autoantibodies	4/37	—	—
Therapy	10/37	—	—
Refractoriness	5/37	—	—
Splenectomised	0/37	—	—

Summary of participant characteristics including demographic data and study design. AITP: autoimmune thrombocytopenia.

**Table 2:** Combinations of oxidative stress and antioxidant capacities in AITP patients and controls. As demonstrated in Table 2, all patients without OS had normal or good AOCs, whereas two healthy controls had reduced AOCs. Overall, 19 controls did not demonstrate OS, in comparison to only nine patients. Interestingly, 14 patients demonstrated strong levels of OS, in comparison to only four healthy controls. However, only 5 of 37 (13.5%) patients demonstrated a pathological combination (strong OS, reduced AOCs), with a higher tendency towards oxidative damage. OS: oxidative stress; AOCs: antioxidant capacities; *n*: number; AITP: autoimmune thrombocytopenia.

Combinations	AITP	Controls
No OS		
Normal or good AOC	*n* = 9	*n* = 17
Reduced AOC	*n* = 0	*n* = 2
Moderate OS		
Normal or good AOC	*n* = 11	*n* = 11
Reduced AOC	*n* = 3	*n* = 3
Strong OS		
Normal or good AOC	*n* = 9	*n* = 3
Reduced AOC	*n* = 5	*n* = 1
