# Supplementary material for: Oxidative Stress Is Predominant in Female but Not in Male Patients with Autoimmune Thrombocytopenia
Source: Oxid Med Cell Longev. 2014 Jan 6;2014:720347. doi: 10.1155/2014/720347 (PMC3941602; doi:10.1155/2014/720347)
Supplement: Supplementary file 1 — Supplement 1 describes a step-by-step schematic overview of the FORT and FORD assays. Supplement 2 entails a questionnaire developed from Micromedical completed by participants which was provided with the FORT and FORD test kits, and the frequency of each response. The questionnaire comprised of four main sections concerning A “general well-being and stress,” B “diet and lifestyle,” C “health and disease,” and D “drug intake”. [file 720347.f1.docx]

**Supplement 1:** Step-by-step schematic overview FORT and FORD assay protocol


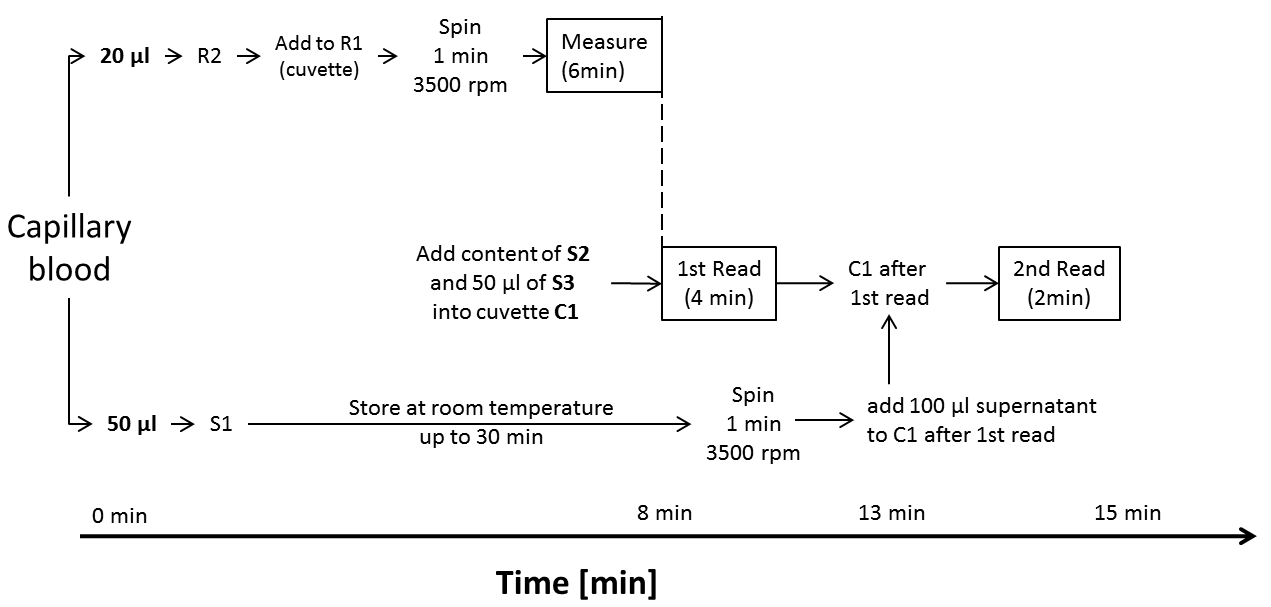


This schematic overview represents the procedure involved in the FORT and FORD assay. Results are usually available within 15 minutes.

**Supplement 2:** Summary of oxidative stress questionnaire responses (Full list)

| **Category** | **Questions** | **Frequency in AITP n (%)** | **Frequency in HD (n / %)** |
| --- | --- | --- | --- |
| ***A) General well-being and stress*** | Do you feel weak? | 25 (67.6 %) | 1 (2.7 %) |
|  | Do you regularly suffer from colds/infections (>3x / year)? | 6 (16.2 %) | 6 (16.2 %) |
|  | Do you feel fully effective/productive? | 26 (70.3%) | 34 (91.9 %) |
|  | Do you feel exhausted? | 18 (48.6) | 1 (2.7 %) |
|  | Are you often stressed? | 24 (64.9%) | 13 (35.1 %) |
| ***B) Diet and lifestyle*** | Do you smoke cigarettes? | 3 (8.1 %) | 9 (24.3 %) |
|  | Are you exposed to passive smoke? | 8 (21.6 %) | 9 (24.3 %) |
|  | Do you consume alcohol on a regular level (>3x/week)? | 8 (21.6 %) | 12 (32.4 %) |
|  | Do you undertake physical activities? (>3x/week) | 18 (48.6 %) | 19 (51.4%) |
|  | Are you frequently exposed to the sun? | 9 (24.3 %) | 10 (27.0%) |
|  | Do you visit a solarium? | 0 (0 %) | 1 (2.7 %) |
|  | Are you a frequent flyer? | 0 (0 %) | 2 (5.4 %) |
|  | Do you spend much time using a computer? | 27 (73.0%) | 27 (73.0%) |
|  | Do you frequently consume fruit? (>3x/week) | 21 (56.8 %) | 15 (40.5 %) |
|  | Do you frequently consume vegetables/salads? (>3x/week) | 18 (48.6 %) | 10 (27.0 %) |
|  | Do you frequently consume fruit/vegetable juices? (>3x/week) | 14 (37.8 %) | 5 (13.5 %) |
|  | Intake of multivitamins/minerals? | 12 (32.4 %) | 8 (21.6 %) |
|  | Intake of radical scavengers? | 2 (5.4 %) | 3 (8.1 %) |
| ***C) health and disease*** | Are you pregnant? | 2 (5.4 %) | 0 (0 %) |
|  | Are you overweight? | 11(29.7 %) | 6 (16.2 %) |
|  | Do you have allergies? | 15 (40.5 %) | 14 (37.8%) |
|  | Do you have diabetes mellitus? | 1 (2.7 %) | 1 (2.7 %) |
|  | Do you have any fat-associated metabolic diseases? | 3 (8.1 %) | 3 (8.1 %) |
|  | Do you have a cardiovascular disease? | 2 (5.4 %) | 0 (0 %) |
|  | Do you have a rheumatic disease? | 1 (2.7 %) | 0 (0 %) |
|  | Do you have a colorectal disease? | 2 (5.4 %) | 0 (0 %) |
|  | Do you have cancer? | 1 (2.7 %) | 0 (0 %) |
|  | Do you have a chronic respiratory disease? | 0 (0 %) | 1 (2.7 %) |
|  | Do you have an amalgam load? | 9 (24.3 %) | 6 (16.2 %) |
|  | Have you been frequently x-rayed within the last 12 months? | 3 (8.1 %) | 3 (8.1 %) |
| ***D) Drug intake*** | Do you take the anti-baby pill? | 2 (5.4 %) | 2 (5.4 %) |
|  | Do you take painkillers? | 5 (13.5 %) | 0 (0 %) |
|  | Do you take hormones? | 3 (8.1 %) | 0 (0 %) |
